# 

**Published:** 1847-10

**Authors:** 


### CONTENTS OF Nº XLVIII

OF THE

### British and Foreign Medical Review.

OCTOBER, 1847.

---

### Part First.—Analytical and Critical Reviews.

---

PAGE

Art. I.—Handbuch der rationellen Pathologie. Von Dr. J. Henle, Professor der Anatomie und Physiologie in Heidelberg. Erster Band. Einleitung und allgemeiner Theil. Zweite unveränderte Auflage . . . . 289

Manual of Rational Pathology. By Dr. J. Henle, Professor of Anatomy and Physiology in Heidelberg. First Volume. Introduction and General Part. Second and unchanged edition.

Idea and Nature of Disease . . . . . 297
General Etiology . . . . . . 298
Locality of Disease, and mode of transition from one organ to another . 304
Course and Events of Disease . . . . . 315

Art. II.—Medico-Chirurgical Transactions. Vol. XXIX . . . 321

Dr. Johnson on the Minute Anatomy and Pathology of Bright's Disease . ib.
Dr. Warren's case of Ligature of the Left Subclavian Artery . . 322
Mr. Vincent on Disease of the Brain . . . . . 323
Mr. Arnott's case of punctured Wound and Ligature of the posterior Tibial Artery . . . . . . ib.
Mr. Paget on imperfectly formed Corpus Callosum, &c. . . ib.
Mr. Hewett on Aneurism . . . . . ib.
Mr. Spitta on Cyanosis . . . . . 324
Mr Rainey on the Arachnoid Membrane of the Brain and Spinal Marrow ib.
Mr. Acton's case of Monstrosity . . . . 325
Mr. Liston on Wounded Arteries . . . . 326
Mr. Dixon's case of a large Tumour in the substance of the Fifth Nerve . 327
Mr. Hutchinson on the Capacity of the Lungs . . . ib.
Mr. Lowdell on Hydatid Cyst . . . . 328
Mr. Curling on Varicocele treated by Pressure . . . ib.
Mr. Busk on congenital Deficiency of one Kidney . . . 329
Dr. Jackson on Derangement of the Structure of the Spleen . . ib.
Mr. Cumming on a Luminous Appearance of the Human Eye . . 330
Mr. Busk on Abscess in the Neck . . . . 331
Mr. Toynbee on the Structure of the Kidney . . . ib.
Dr. Thomson on the relation between the Constituents of Food and the Systems of Animals . . . . . 332

§

PAGE

ART. III.—1. On the Pathology and Treatment of Dysentery, being the Gulstonian Lectures, delivered at the College of Physicians in February, 1847. By WILLIAM BALY, M.D., Physician to the Milbank Prison, and Lecturer on Forensic Medicine at St. Bartholomew's Hospital. From the Medical Gazette . . . . . . . . 333

2. Observations on the History and Treatment of Dysentery and its Combinations, &c. &c. By WILLIAM HARTY, M.D., Physician to the King's Hospital and to the Prisons of Dublin. Second Edition . . . ib.

3. Notes on the Pathology and Treatment of Dysentery, as observed in the European General Hospital at Bombay during the five years from July, 1838, to July, 1843. By C. MOREHEAD, M.D. (Transactions of the Medical and Physical Society of Bombay, No. VII. Presented April, 1845) . . ib.

4. A Dictionary of Practical Medicine. By JAMES COPLAND, M.D., F.R.S. Vol. I. Art. Dysentery . . . . . . ib.

Simple a-pyrectic non-contagious Dysentery . . . 334
Combined and complicated Dysentery . . . . 345

ART. IV.—1. Saggi di Clinica Medica, basati specialmente sopra le Osservazioni della Clinica del Professor MAURIZIO BUFALINI. Compilati dal Dottor F. BINI, Ajuto alla Clinica Medica della Scuola di Complemento e Perfezionamento nell' I.E.R. Archispedale di S. Maria Nuova di Firenze, e dall Dottor GHINOZZI . . . . . . . 369

Essays on Clinical Medicine, founded especially on Observations of the Clinique of Professor M. BUFALINI. Compiled by Dr. F. BINI, Clinical Assistant of the School of Completion in the large Hospital of Florence, and by Dr. C. GHINOZZI.

2. La Clinica Medica del Professore G. A. GIACOMINI. Esposizione compendiata del Dottore G. B. MUGNA . . . . . ib.

A short Dissertation on the Clinical Medicine of Professor G. A. GIACOMINI. By Dr. G. B. MUGNA.

ART. V.—On Cataract, Artificial Pupil, and Strabismus. By F. H. BRETT, Esq., M.D., F.R.C.S. . . . . . . 364

ART. VI.—Den Pathologiske Anatomie's Svar paa Spörgsmaalet: Hoad er Cancer? Ved ADOLPH HANNOVER, Lic. Med. . . . . 373

The Response of Pathological Anatomy to the Question: "What is Cancer?" By ADOLPH HANNOVER, Licentiate of Medicine. With a Lithographed Plate.

ART. VII.—Della Mortalità e dimora media dei malati nello Spedale Maggiore di Milana dal 1811 al 1844, ed in quello dei RR. Fatebenefratelli dal 1604 al 1844, coi prospetti del calcolo compelessivo sopra 784,000 infermi. Memoria del Medico-Statista Dottore GUISEPPE FERRARIO, di Milano . . 379

On the Mortality and Mean Length of Abode of the Patients in the Large Hospital of Milan from 1811 to 1844, and in that of the Brothers of Charity from 1604 to 1844, &c. A Memoir, by Dr. G. FERRARIO, Medical Statist, of Milan.

ART. VIII.—Ueber Schädel-bildung, zur festeren Begrundung der Menschen-rassen. Von Prof. Dr. A. ZENNE . . . . . 381

On the Form of the Skull as supplying a Distinctive Character of the Varieties of Mankind. By Professor ZENNE, M.D.

PAGE

ART. IX.—1. Théorie Expérimentale de la Formation des Os. Par P. FLOURENS, Secrétaire perpétuel de l'Académie Royale des Sciences, Professeur de Physiologie comparée au Muséum d'Histoire Naturelle de Paris, &c. &c. . 386

Experimental Theory of the Formation of Bone. By P. FLOURENS, Perpetual Secretary of the Royal Academy of Sciences, Professor of Comparative Physiology in the Museum of Natural History of Paris, &c. &c.

2. Elements of Anatomy. By JONES QUAIN, M.D. Fifth Edition. Edited by RICHARD QUAIN and WILLIAM SHARPEY, M.D., Professors of Anatomy and Physiology, University College, London . . . ib.

3. Cyclopædia of Anatomy and Physiology. Edited by ROBERT B. TODD, M.D., F.R.S., Professor of Physiology and of General and Morbid Anatomy in King's College, London, &c. &c. Article, Osseous Tissue. By JOHN TOMES, Surgeon-Dentist to the Middlesex Hospital . . . ib.

ART. X.—Der Winterschlaf nach seinen Erscheinungen im Thierreich dargestellt von Dr. H. C. L. BARKOW, ordentlichen Professor der Medicin und Director des Anatomie-Instituts an der königlichen Universität zu Breslau . 408

Hybernation considered in its Phenomena throughout the Animal Kingdom. By Dr. H. C. L. BARKOW, Ordinary Professor of Medicine and Director of the Anatomical Establishment in the Royal University of Breslau.

ART. XI.—A Treatise on the Structure, Diseases, and Injuries of the Blood-Vessels. By EDWARDS CRISP . . . . . . 410

ART. XII.—Om Torsk hos Barn. Af Dr. FR. TH. BERG, Ofverläkare vid Stockholms Allm. Barnhus, &c. . . . . . . . 422

On Thrush in Children. By Dr. FR. TH. BERG, Physician in Chief to the General Children's Hospital in Stockholm, &c.

ART. XIII.—1. The Phytologist, a Popular Botanical Miscellany. Conducted by GEORGE LUXFORD, A.L.S., F.B.S.E. . . . . 436

2. The Zoologist, a Popular Miscellany of Natural History. Conducted by EDWARD NEWMAN, F.L.S., Z.S., &c. . . . . ib.

ART. XIV.—1. Researches into the Physical History of Mankind. By JAMES COWLES PRICHARD, M.D., F.R.S., M.R.I.A., Corresponding Member of the National Institute of France, &c. &c. . . . . . 441

2. The Natural History of Man; comprising Inquiries into the Modifying Influence of Physical and Moral Agencies on the different Tribes of the Human Family. By JAMES COWLES PRICHARD, M.D., F.R.S., M.R.I.A., Corresponding Member of the National Institute of France, &c. &c. . ib.

ART. XV.—A Treatise on Diseases of the Air-passages: comprising an Inquiry into the History, Pathology, Causes, and Treatment of those Affections of the Throat called Bronchitis, Chronic Laryngitis, Clergyman's Sore Throat, &c. By HORACE GREEN, A.M., M.D., formerly President and Professor of the Theory and Practice of Medicine in the Castleton Medical College, Vice-President of the New York Medical and Surgical Society, and Honorary Member of the Philadelphia Medical Society, &c. &c . . . 482

ART. XVI.—On Tumours of the Uterus and its Appendages. (Jacksonian Prize Dissertation.) By THOMAS STAFFORD LEE, M.R.C.S.E., Fellow of the Medico-Chirurgical Society, &c. &c. . . . . . 506

PAGE

ART. XVII.—Proceedings of the World's Temperance Convention, held in London, August 4th, 1846, and following Days. With the Papers laid before the Convention, Letters read, Statistics, and General Information presented, &c. 515

ART. XVIII.—1. Contributions to Clinical Medicine. On Pericarditis. In Lancet from May 17, 1845, to October 31, 1846. By JOHN TAYLOR, M.D., Physician to the Huddersfield Infirmary, late Professor of Clinical Medicine in University College, London, &c. . . . . . 548

2. On some of the Causes of Pericarditis, especially Acute Rheumatism and Bright's Disease of the Kidneys, &c. Medico-Chirurgical Transactions, Vol. XXVIII, 1846. By JOHN TAYLOR, M.D., &c. . . . ib.

ART. XIX.—The Human Brain: its Structure, Physiology, and Diseases. With a Description of the Typical Forms of Brain in the Animal Kingdom. By SAMUEL SOLLY, F.R.S., Senior Assistant-Surgeon to St. Thomas's Hospital, and Lecturer on Clinical Surgery, &c. &c. . . . . 572

ART. XX.—Observations on the Treatment of Lateral Curvature of the Spine, &c. By EDWARD F. LONSDALE, Fellow of the Royal College of Surgeons, &c. &c. 578

ART. XXI.—On Dyspepsia; with Remarks, submitted in support of the Opinion that the proximate Cause of this and of all other Diseases affecting the General System is Vitiation of the Blood. By JOHN BURDETT STEWARD, M.D., Fellow of the Royal College of Physicians . . . 579

---

The late Dr. ANDREW COMBE . . . . . . 581

Postscript to No. XLVIII of the British and Foreign Medical Review . . 585

INDEX, TITLE, &c.

